# Safety of Combined Division vs Separate Division of the Splenic Vein in Patients Undergoing Distal Pancreatectomy: A Noninferiority Randomized Clinical Trial

**DOI:** 10.1001/jamasurg.2021.0108

**Published:** 2021-03-03

**Authors:** Suguru Yamada, Tsutomu Fujii, Fuminori Sonohara, Manabu Kawai, Kazuto Shibuya, Ippei Matsumoto, Kengo Fukuzawa, Hideo Baba, Takeshi Aoki, Michiaki Unno, Sohei Satoi, Yoji Kishi, Etsuro Hatano, Kenichiro Uemura, Akihiko Horiguchi, Masayuki Sho, Yutaka Takeda, Toshio Shimokawa, Yasuhiro Kodera, Hiroki Yamaue

**Affiliations:** 1 Department of Gastroenterological Surgery (Surgery II), Nagoya University Graduate School of Medicine, Tsurumai-cho, Showa-ku, Nagoya, Japan; 2 Department of Surgery and Science, Faculty of Medicine, Academic Assembly, University of Toyama, Sugitani, Toyama, Japan; 3 Second Department of Surgery, Wakayama Medical University, Kimiidera, Wakayama, Japan; 4 Department of Surgery, Kindai University Faculty of Medicine, Ono-higashi, Osaka-Sayama, Japan; 5 Department of Surgery, Oita Red Cross Hospital, Chiyo-machi, Oita, Japan; 6 Department of Gastroenterological Surgery, Kumamoto University Graduate School of Medical Science, Honjo, Chuo-ku, Kumamoto, Japan; 7 Department of Gastroenterological and General Surgery, Showa University School of Medicine, Hatanodai, Shinagawa-ku, Tokyo, Japan; 8 Division of Hepato-Biliary Pancreatic Surgery, Tohoku University Graduate School of Medicine, Seiryo-cho, Aoba-ku, Sendai, Japan; 9 Department of Surgery, Kansai Medical University, Shinmachi, Hirakata, Japan; 10 Department of Surgery, National Defense Medical College, Namiki, Tokorozawa, Saitama, Japan; 11 Department of Gastroenterological Surgery, Hyogo College of Medicine, Mukogawa, Nishinomiya, Hyogo, Japan; 12 Department of Surgery, Hiroshima University Graduate School of Biomedical and Health Sciences, Kasumi, Minami-ku, Hiroshima, Japan; 13 Department of Gastroenterological Surgery, Fujita Health University Bantane Hospital, Otobashi, Nakagawa-ku, Nagoya, Japan; 14 Department of Surgery, Nara Medical University School of Medicine, Shijo-cho, Kashihara, Nara, Japan; 15 Department of Surgery, Japan Organization of Occupational Health and Safety, Kansai Rosai Hospital, Inabaso, Amagasaki, Japan; 16 Clinical Study Support Center, Wakayama Medical University, Kimiidera, Wakayama, Japan

## Abstract

**Importance:**

In distal pancreatectomy (DP), the splenic vein is isolated from the pancreatic parenchyma prior to being ligated and divided to prevent intra-abdominal hemorrhage from the splenic vein stump with pancreatic fistula (PF). Conversely, dissecting the splenic vein with the pancreatic parenchyma is easy and time-saving.

**Objective:**

To establish the safety of combined division of the splenic vein compared with separate division of the splenic vein.

**Design, Setting, and Participants:**

This study was designed as a multicenter prospective randomized phase 3 trial. All results were analyzed using the modified intent-to-treat set. Patients undergoing DP for pancreatic body and tail tumors were eligible for inclusion. Patients were randomly assigned between August 10, 2016, and July 30, 2019.

**Interventions:**

Patients were centrally randomized (1:1) to either separate division of the splenic vein or combined division of the splenic vein.

**Main Outcomes and Measures:**

The primary end point was the incidence of grade B/C PF, and the incidence of intra-abdominal hemorrhage was included as one of the secondary end points.

**Results:**

A total of 318 patients were randomly assigned, and 2 patients were excluded as ineligible. Of the 316 remaining patients, 150 (50.3%) were male. The modified intent-to-treat population constituted 159 patients (50.3%) in the separate division group and 157 patients (49.7%) in the combined division group. In the modified intent-to-treat set, the proportion of grade B/C PF in the separate division group was 27.1% (42 of 155) vs 28.6% (44 of 154) in the combined division group (adjusted odds ratio, 1.108; 95% CI, 0.847-1.225; *P* = .047), demonstrating noninferiority of the combined division of the splenic vein against separate division. The incidence of postoperative intra-abdominal hemorrhage in the 2 groups was identical at 1.3%.

**Conclusions and Relevance:**

This study demonstrated noninferiority of the combined division of the splenic vein compared with separate division of the splenic vein regarding safety. Thus, isolating the splenic vein from the pancreatic parenchyma is deemed unnecessary.

**Trial Registration:**

ClinicalTrials.gov Identifier: NCT02871804

## Key Points

**Question:**

In distal pancreatectomy, is combined division of the splenic vein safe compared with separate division of the splenic vein?

**Findings:**

In this noninferiority randomized clinical trial, the proportion of grade B/C pancreatic fistula in the separate division group was 27.1% vs 28.6% in the combined division group, demonstrating noninferiority of the combined division of the splenic vein against separate division.

**Meaning:**

The safety of combined division of the splenic vein in distal pancreatectomy was established, such that the approach could be recommended with more confidence.

## Teaser

This noninferiority randomized clinical trial aims to establish the safety of combined division of the splenic vein compared with separate division of the splenic vein.

## Introduction

Generally, distal pancreatectomy (DP) involves not only mandatory dissection of the pancreas but also dissection of the splenic artery and vein. During this surgical procedure, the splenic vein is often isolated from the pancreatic parenchyma prior to being ligated and divided. This aims to prevent intra-abdominal hemorrhage from the stump of the splenic vein with pancreatic fistula (PF), which is commonly observed after DP (8.6%-42.3%).^1,2,3,4,5,6,7,8,9,10,11,12,13^ On the other hand, intra-abdominal hemorrhages are reportedly caused by PF or other reasons in 1% to 8% of patients who undergo DP.^2,6,9^

More recently, the widespread use of laparoscopy in DP has led to increased use of mechanical staplers to dissect the pancreas. Under such circumstances, dissecting the splenic vein with the pancreatic parenchyma is generally preferred owing to its apparent technical simplicity, and this method has actually become the standard at some institutions.^14,15^ However, PF occurring after this type of resection is of deep concern to surgeons because of the risk of 1% to 8% intra-abdominal bleeding from the stump of the splenic vein,^2,6,9^ which could then be immersed in effusion-rich pancreatic juice.

To date and to our knowledge, no scientific evidence concerning the safety of this useful but potentially hazardous surgical procedure has been reported. Therefore, we conducted this multicenter, randomized, phase 3 trial, Combined Resection vs Separated Resection After Mobilization of the Splenic Vein During Distal Pancreatectomy (COSMOS-DP), to establish the safety of this procedure so that it can be recommended with more confidence.

## Methods

### Study Design

The COSMOS-DP trial study protocol was published and internationally registered^17^ and is also available in Supplement 1. COSMOS-DP was designed as a multicenter (45 institutions across Japan) prospective randomized phase 3 trial (eTable 1 in Supplement 2). The aim of the study was to establish noninferiority in terms of safety of dividing the splenic vein with the pancreatic parenchyma compared with that of the conventional technique of isolating the vein from the pancreas before ligation and division. The comparison was made during DP in which mechanical staplers were used. We hypothesized that dividing the splenic vein with the pancreatic parenchyma by mechanical stapler could be similarly safe to the conventional technique of isolating the vein from the pancreas before ligation and division in terms of the incidence of grade B/C PF. This study was conducted according to the Declaration of Helsinki^16^ and the ethical guidelines for medical and health research involving human subjects in Japan. Written informed consent was obtained from all the enrolled patients. Ethical approval for this study was obtained from the institutional review board of each institution.

### Patients

Patients undergoing open or laparoscopic DP for pancreatic body and tail cancer, intraductal papillary mucinous neoplasm, neuroendocrine tumors, mucinous cystic neoplasm, or metastatic pancreatic tumors were eligible for inclusion in this study. Simultaneous division of the pancreatic parenchyma and splenic vein in 1 session was deemed possible by evaluating preoperative imaging study findings. Briefly, the estimated pancreatic cut end was assumed to be at the left side from the left border of the portal vein in all patients. A detailed overview of the eligibility criteria is provided in eTable 2 in Supplement 2.

### Randomization and Interventions

We used a central randomization and registration system (1:1). After assessing patients for eligibility, they were centrally randomized to either arm A (separate division of the splenic vein) or arm B (combined division of the splenic vein) before surgery between August 10, 2016, and July 30, 2019. Following randomization, patients were stratified according to the surgical approach (open or laparoscopic), institution, and thickness of the pancreatic parenchyma (<15 mm or ≥15 mm). We used Pocock and Simon minimization method for random assignments and the Mersenne Twister method for random number generation.

A linear stapler (Endo GIA Reinforced Reload with Tri-Staple Technology [black cartridge]; Covidien) was used in all patients. The pancreatic parenchyma was compressed with the stapler at the planned line of resection for more than 5 minutes before transection was performed. For the patients in arm A, the splenic vein was isolated from the pancreatic parenchyma and dissected after ligation (eFigure 1 in Supplement 2). For those in arm B, the splenic vein was transected concurrently with the pancreatic parenchyma using the aforementioned stapler (eFigure 2 in Supplement 2).

To confirm that the surgical procedures were conducted as allocated at the time of central judgment, 2 photographs (before and after pancreatic transection) were taken for all patients. Central judgment was conducted biannually for all registered patients. At that time, the photographs were reviewed by 2 members of the committee.

### Outcomes

The primary end point was the incidence of grade B/C PF. The secondary end points were outcome measures related to surgery, such as the operative time, blood loss volume, hemostasis of the staple line, integrity of the staple line, incidence of pancreatic injury, need for additional sutures to securely close the pancreatic stump, drainage duration, postoperative hospital stay duration, and incidence of conversion from laparoscopic surgery to open surgery. The outcome measures related to complications were the incidence of all grades of PF, incidence of grade C PF, incidence of intra-abdominal hemorrhage, incidence of all complications, mortality, and the incidence of splenic vein thrombosis (1 and 6 months after surgery), which were also included as secondary end points.

A complication was defined as an event occurring within 6 months after surgery. The definition of PF was according to the International Study Group of Postoperative Pancreatic Fistula classification,^18^ and the definition of delayed gastric emptying was according to the International Study Group of Pancreatic Surgeons classification.^19^ The definition of intra-abdominal bleeding was according to the International Study Group of Pancreatic Surgeons classification,^20^ and the definitions of other postoperative complications were according to the Clavien-Dindo classification.^21^

### Statistical Analysis

Diener et al^2^ reported that the incidence of grade B/C PF in separate division was 11.5%; therefore, we determined that an incidence rate of grade B/C PF more than 10% higher than that in the combined division group (10%) would be clinically problematic. We set the noninferiority margin at 9%, and the incidence of grade B/C PF in this study was set to 10%. For an assumed PF incidence rate of 10% with a noninferiority margin of 9%, the difference in the allowable PF incidence rate between arms A and B was 0.09. The odds of an expected incidence rate of grade B/C PF of 0.111 in arm A and those in arm B (noninferiority margin + expected incidence rate of grade B/C PF in arm A) form an incidence rate of 0.2346, so the odds ratio is 2.11. When the statistical analysis was performed for a significance level of α = .05 (1-sided) in a noninferiority design, 138 patients were required per arm, with a power 100 (1 − β) of more than 80%, assuming that a small number of patients might be deemed ineligible and thus might be excluded from the analysis. Furthermore, as approximately 5% of the patients were expected to be ineligible for surgery as indicated by the laparotomy or laparoscopic findings, the sample size was eventually increased to 304 patients (152 patients per arm).

In this study, an interim analysis was performed once. The multiplicity of tests in the interim and final analyses were adjusted using Lan-DeMets α consumption function to keep the overall test 1-sided α error at 5.0%. The difference in the incidence rate of grade B/C PF between arms was evaluated using the Farrington-Manning test with a noninferiority margin of 9%. For the α consumption function, we used the O’Brien-Fleming type. During this trial, the interim analysis was conducted on December 14, 2018, after recruiting 187 study patients. It revealed the adequacy of this study and did not affect the statistical testing of the noninferiority hypothesis. The Data and Safety Monitoring Committee independently reviewed and recommended continuation of patient recruitment up to the initially planned sample size.

For evaluation of the primary end point, we applied the Farrington-Manning test with a noninferiority margin of 9%. The differences of proportions for each group and exact 2-sided 90% CIs were used to evaluate the noninferiority margin of 0.09, and we also calculated the exact 2-sided 95% CI for reference. Adjusted odds ratio with 95% CIs of the incidence of grade B/C PF between arms A and B was calculated using multiple logistic regression analysis with the surgical approach and thickness of the pancreatic parenchyma as covariates. The incidence rates of grade B/C PF for each group were summarized as frequencies and incidence proportions with Clopper-Pearson exact 95% CIs.

As secondary end points, we compared binary variables with Fisher exact test and continuous variables with the Mann-Whitney *U* test. For comparison of efficacy secondary end points, statistical tests were 2 sided with a significance level of .05 because their results for secondary end points should be interpreted as explanatory. For safety analyses, we summarized the incidence of adverse events using rates and exact 2-sided 95% CIs. In addition, the incidence of adverse events was evaluated using Fisher exact test with 2-sided significance level of .05.

All results were analyzed using the modified intent-to-treat set, which included all patients except for those deemed ineligible after registration. We performed a similar analysis using the per-protocol set as a reference. All analyses were performed using R version 4.0.0 (R Foundation).

## Results

### Patients

A total of 318 patients (median of 4 patients enrolled from each hospital; range, 1-64) were randomly assigned to the separate division group (arm A; 159 patients) and the combined division group (arm B; 159 patients) (Figure). Of these, 2 patients were found to be ineligible after enrollment and were excluded from the subsequent analyses. The modified intent-to-treat population constituted 316 patients (159 patients [50.3%] in the separate division group and 157 patients [49.7%] in the combined division group), of whom 39 patients were not treated according to the protocol because of contraindications for resection, technical problems, exclusion criteria, or other reasons. Eleven patients whose procedures were converted from combined division to separate division were excluded. Finally, the remaining per-protocol population constituted 146 patients (52.3%) in the separate division group and 133 patients (47.7%) in the combined division group.

**Figure.**
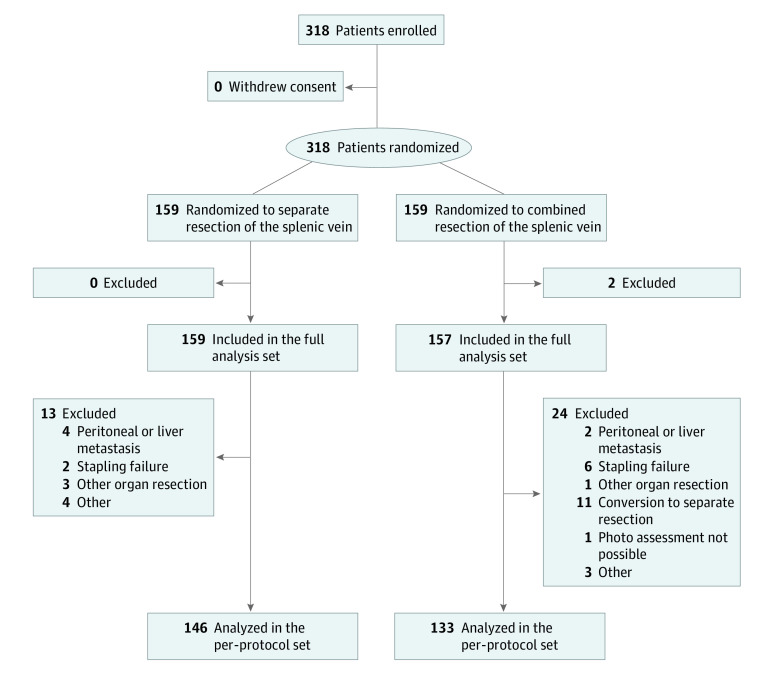
Study Flowchart for the Combined Resection vs Separated Resection After Mobilization of the Splenic Vein During Distal Pancreatectomy (COSMOS-DP) Trial

### Baseline Clinical Characteristics

Preoperative patients’ baseline clinical characteristics between the groups based on the modified intent-to-treat set are presented in Table 1. Patients’ backgrounds, including the thickness of the pancreas and main pancreatic duct diameter, and preoperative laboratory data were well balanced between the groups. Operative and postoperative patients’ baseline clinical characteristics between the groups based on the modified intent-to-treat set are presented in Table 2. Regarding operative findings, there was no difference in the pancreatic texture between the groups. Regarding the postoperative laboratory data, there were no differences between the groups for all parameters, including amylase concentration in the drainage fluid.

**Table 1.** Preoperative Patients’ Baseline Clinical Characteristics in the Modified Intent-to-Treat Set

Characteristic	Division of the splenic vein			
	Separate		Combined	
	No. (%)	Missing value	No. (%)	Missing value
Total No.	159	NA	157	NA
Age, median (range), y	70 (20-89)	0	65 (22-89)	0
Sex				
Male	84 (52.8)	0	75 (47.8)	0
Female	75 (47.2)		82 (52.2)	
BMI, median (range)	21.7 (14.5-38.2)	0	22.0 (14.9-37.5)	0
Performance status				
0	133 (83.6)	0	141 (89.8)	0
1	26 (16.4)		16 (10.2)	
Diabetes				
No	115 (72.3)	0	123 (78.3)	0
Yes	4 (27.7)		34 (21.7)	
Steroid use				
No	156 (98.1)	0	154 (98.1)	0
Yes	3 (1.9)		3 (1.9)	
Anticoagulant use				
No	138 (86.8)	0	140 (89.2)	0
Yes	21 (13.2)		17 (10.8)	
Cardiovascular disease				
No	141 (88.7)	0	136 (86.6)	0
Yes	18 (11.3)		21 (13.4)	
Radiation				
No	156 (98.1)	0	155 (98.7)	0
Yes	3 (1.9)		2 (1.3)	
Chemotherapy				
No	151 (95.0)	0	152 (96.8)	0
Yes	8 (5.0)		5 (3.2)	
Pancreatic thickness, median (range), mm	13.5 (5.7-32.1)	0	14.0 (4.9-26.0)	0
Main pancreatic duct diameter, median (range), mm	2.1 (1.0-12.5)	0	2.1 (0.3-8.6)	0
Distance between the left border of the portal vein and the estimated pancreatic cut end, median (range), mm	13.2 (0.0-85.0)	0	16.0 (0.0-75.0)	0
Primary disease				
Pancreatic cancer	71 (44.7)	0	62 (39.5)	0
Intraductal papillary mucinous neoplasm	32 (20.1)		24 (15.3)	
Pancreatic neuroendocrine neoplasm	17 (10.7)		24 (15.3)	
Mucinous cystic neoplasm	14 (8.8)		24 (15.3)	
Other	25 (15.7)		23 (12.6)	
Laboratory data, median (range)				
White blood cell count, /μL	5400 (2000-11 800)	0	5500 (2730-14 430)	0
Hemoglobin, g/dL	13.4 (9.2-16.1)	0	13.2 (9.8-17.7)	0
Platelet count, ×10^3^/µL	213 (91-985)	0	215 (61-397)	0
Total bilirubin, mg/dL	0.7 (0.20-1.83)	0	0.6 (0.20-1.80)	0
Creatinine, mg/dL	0.71 (0.43-1.65)	0	0.72 (0.44-1.60)	0
Albumin, g/dL	4.1 (2.3-5.0)	0	4.1 (3.0-5.3)	0
Serum amylase, U/L	79 (23-775)	0	77 (30-362)	2
Hemoglobin A_1c_, % of total hemoglobin	6.0 (3.9-11.2)	2	5.9 (4.9-10.8)	2
Modulator for allocation				
Operative procedure				
Open	73 (45.9)	0	69 (43.9)	0
Laparoscopic	86 (54.1)		88 (56.1)	
Thickness of the pancreatic parenchyma, mm				
≥15	104 (65.4)	0	100 (63.7)	0
<15	55 (34.6)		57 (36.3)	

Abbreviations: BMI, body mass index (calculated as weight in kilograms divided by height in meters squared); NA, not applicable.

SI conversion factors: To convert albumin to g/L, multiply by 10; amylase to µkat/L, multiply by 0.0167; bilirubin to µmol/L, multiply by 17.104; creatinine to µmol/L, multiply by 88.4; hemoglobin to g/L, multiply by 10; hemoglobin A_1c_ to proportion of total hemoglobin, multiply by 0.01; platelet count to ×10^9^/L, multiply by 1; white blood cell count to ×10^9^/L, multiply by 0.001.

**Table 2.** Operative and Postoperative Patients’ Baseline Clinical Characteristics in the Modified Intent-to-Treat Set

Characteristic	Division of the splenic vein			
	Separate		Combined	
	No. (%)	Missing value	No. (%)	Missing value
Total No.	159	NA	157	NA
**Operative findings**				
Operation				
Open	70 (45.2)	4	67 (43.5)	3
Laparoscopic	85 (54.8)		87 (56.5)	
Conversion	12 (14.1)	0	12 (13.8)	0
Procedure				
Distal pancreatectomy	151 (97.4)	NA	153 (99.4)	NA
DP-CAR	1 (0.6)		0	
Warshaw	3 (1.9)		1 (0.6)	
Lymph node dissection				
D0	21 (13.5)	4	19 (12.3)	3
D1	64 (41.3)		75 (48.7)	
D2	70 (45.2)		60 (39.0)	
Operative time, median (range), min	248 (106.0-574.0)	4	251.5 (80.0-612.0)	3
Blood loss, median (range), mL	105 (0-1612.0)	4	100 (0-3170.0)	3
Blood transfusion				
No	150 (96.8)	4	148 (94.3)	3
Yes	5 (3.2)		6 (3.8)	
Pancreatic texture				
Soft	131 (84.5)	4	132 (85.7)	3
Hard	24 (15.5)		22 (14.3)	
Cartridge type				
Black	151 (98.7)	6	151 (98.7)	4
Purple	2 (1.3)		2 (1.3)	
Cartridge No.				
1	149 (98.0)	7	152 (99.3)	4
2	3 (2.0)		1 (0.7)	
Status of reinforcement sheet after resection				
Coated	141 (92.8)	7	147 (96.1)	4
Not coated (damaged/defect)	11 (7.2)		6 (3.9)	
**Stapling findings**				
Malfunctioning stapler				
No	151 (98.7)	6	153 (100.0)	4
Yes	2 (1.3)		0	
Hemostasis of the staple line				
None	143 (94.1)	7	138 (90.2)	4
Compression/coagulation	9 (5.9)		14 (9.2)	
Suture	0		1 (0.7)	
Incidence of pancreatic injury				
None	149 (98.0)	7	144 (94.1)	4
Repair	3 (2.0)		8 (5.2)	
Reresection	0		1 (0.7)	
Additional suturing of the stump				
No	150 (98.7)	7	147 (96.1)	4
Yes	2 (1.3)		6 (3.9)	
Intraoperative adverse event				
No	151 (97.4)	4	148 (96.1)	3
Yes	4 (2.6)		6 (3.9)	
**Postoperative laboratory data, median (range)**				
White blood cell count, /μL				
Postoperative d				
1	11 200 (4640-20 860)	4	11 745 (5600-20 600)	3
3	11 830 (3540-25 370)	6	13 240 (4910-29 590)	3
7	7580 (2900-24 500)	14	8240 (4000-25 110)	17
Hemoglobin, g/dL				
Postoperative d				
1	11.7 (7.4-14.8)	4	11.9 (8.3-15.8)	3
3	11.5 (6.5-15.6)	6	11.3 (8.0-15.9)	3
7	11.6 (8.2-15.1)	14	11.6 (7.2-14.9)	17
Albumin, g/dL				
Postoperative d				
1	3.1 (2.1-3.9)	4	3.1 (2.2-3.9)	3
3	2.9 (2.0-4.0)	6	2.8 (1.7-3.7)	3
7	3.1 (2.2-4.2)	15	3.1 (2.0-3.8)	17
Serum amylase, U/L				
Postoperative d				
1	173 (27-2425)	5	174 (20-2265)	3
3	51 (14-683)	6	50 (17-452)	3
7	59 (15-371)	16	54 (11-229)	18
C-reactive protein, mg/dL				
Postoperative d				
1	0.57 (0.02-1.69)	4	0.57 (0.03-1.56)	3
3	1.34 (0.08-3.51)	6	1.66 (0.13-3.84)	3
7	0.36 (0.04-2.08)	14	0.40 (0.06-2.76)	17
Postoperative findings, median (range)				
Drain amylase concentration, IU/L				
Postoperative d				
1	4516 (54-39 713)	4	5101 (99-55 352)	3
3	834 (27-107 780)	5	913 (37-12 847)	7
Drainage duration, d	6 (1-175)	4	6 (1-118)	3
Postoperative stay, d	16 (4-101)	4	16 (6-84)	3
**Postoperative complications**				
Pancreatic fistula				
None	62 (40.0)	4	61 (39.6)	4
All	93 (60.0)		93 (60.4)	
Grade A	51 (32.9)		49 (31.8)	
Grade B	41 (26.5)		43 (27.9)	
Grade C	1 (0.6)		1 (0.6)	
Delayed gastric emptying				
None	153 (98.7)	4	151 (98.1)	4
All	2 (1.3)		3 (1.9)	
Grade A	2 (1.3)		3 (1.9)	
Grade B	0		0	
Grade C	0		0	
Intra-abdominal hemorrhage				
None	153 (98.7)	4	152 (98.7)	4
All	2 (1.3)		2 (1.3)	
Grade A	0		0	
Grade B	0		1 (0.6)	
Grade C	2 (1.3)		1 (0.6)	
Any complications				
No	49 (31.4)	3	53 (34.4)	4
Yes	107 (68.6)		101 (65.6)	
Mortality				
No	155 (100.0)	4	154 (100.0)	4
Yes	0		0	
Splenic vein				
Thrombosis at 1 mo				
No	139 (97.2)	16	133 (93.7)	15
Yes	4 (2.8)		9 (6.3)	
Obstruction at 1 mo				
No	138 (96.5)	16	137 (96.5)	15
Yes	5 (3.5)		5 (3.5)	
Thrombosis/obstruction at 1 mo				
No	135 (94.4)	16	129 (90.8)	15
Yes	8 (5.6)		13 (9.2)	
Thrombosis at 6 mo				
No	147 (99.3)	11	134 (97.8)	20
Yes	1 (0.7)		3 (2.2)	
Obstruction at 6 mo				
No	145 (98.0)	11	133 (97.1)	20
Yes	3 (2.0)		4 (2.9)	
Thrombosis/obstruction at 6 mo				
No	144 (97.3)	11	132 (96.4)	20
Yes	4 (2.7)		5 (3.6)	

Abbreviations: DP-CAR, distal pancreatectomy with celiac axis resection; NA, not applicable.

SI conversion factors: To convert albumin to g/L, multiply by 10; amylase to µkat/L, multiply by 0.0167; C-reactive protein to mg/L, multiply by 10; hemoglobin to g/L, multiply by 10; white blood cell count to ×10^9^/L, multiply by 0.001.

### Primary End Point

The primary end point of the COSMOS-DP trial was the incidence of grade B/C PF, which is presented in Table 3. In the modified intent-to-treat set, the proportion of grade B/C PF in the separate division group was 27.1% (42 of 155), whereas that in the combined division group was 28.6% (44 of 154) (adjusted odds ratio, 1.108; 95% CI, 0.847-1.225; *P* = .047). Similarly, the proportion of grade B/C PF in the separate division group in the per-protocol set was 27.4% (40 of 146) and that in the combined division group was 27.1% (36 of 133) (adjusted odds ratio, 1.003; 95% CI, 0.827-1.217; *P* = .03). Thus, the Farrington and Manning test (noninferiority margin = 0.09) demonstrated the noninferiority of combined division of the splenic vein against separate division regarding the incidence of grade B/C PF.

**Table 3.** Primary End Point Data for the Modified Intent-to-Treat (ITT) Set and Per-Protocol Set

Primary end point	No.	Pancreatic fistula		Difference of proportions (95% CI)	Adjusted odds ratio (95% CI)	*P* value
		Grade B/C	Proportion (95% CI), %			
Modified ITT set						
Separate division	155	42	27.1 (20.3 to 34.8)	−0.014 (−0.114 to 0.085)	1.108 (0.847 to 1.225)^a^	.047^b^
Combined division	154	44	28.6 (21.6 to 36.4)			
Per-protocol set						
Separate division	146	40	27.4 (20.3 to 35.4)	0.003 (−0.102 to 0.107)	1.003 (0.827 to 1.217)^a^	.03^b^
Combined division	133	36	27.1 (19.7 to 35.5)			

^a^ Adjusted odds ratios were calculated using logistic regression analysis with surgery (open/laparoscopic) and pancreatic thickness (≥15 mm/<15 mm) as covariates.

^b^ Farrington and Manning test (noninferiority margin = 0.09).

### Secondary End Points

Secondary end point results for the modified intent-to-treat set are shown in Table 4. There were no differences in operative time and blood loss between the groups. The percentage of patients requiring hemostasis of the staple line after pancreatic resection was 9.8% (15 of 154) in the combined division group and 5.9% (9 of 155) in the separate division group. Similarly, the incidence of pancreatic injury (crush injury or laceration) was higher in the combined division group vs the separate division group (5.9% [9 of 154] vs 2.0% [3 of 155], respectively); however, the difference was not statistically significant. Postoperative intra-abdominal hemorrhage was observed in 1.3% of the patients in each group (2 of 154 vs 2 of 155). The hemorrhage from the stump of splenic artery was observed in 1 patient in each group, and the remaining were the hemorrhage from the gastric wall and abdominal wall, respectively. There was no patient who encountered the hemorrhage from the stump of splenic vein in this study. These events were managed adequately and did not result in mortality. Postoperative splenic vein thrombosis was observed in 9.2% of the patients in the combined division group (13 of 154) and 5.6% of the patients in the separate division group (8 of 155), but again, the difference did not reach statistical significance. The similar analytical results for the per-protocol set are presented in eTable 3 in Supplement 2.

**Table 4.** Secondary End Point Data for the Modified Intent-to-Treat Set

Secondary end point	No. (%)		OR (95% CI)	*P* value
	Separate division (n = 155)	Combined division (n = 154)		
Operative time, median (range), min	248 (106-574)	252 (80-612)	NA	.71^a^
Blood loss, median (range), mL	105 (0-1612)	100 (0-3170)	NA	.65^a^
Hemostasis of the staple line				
None	143 (94.1)	138 (90.2)	NA	.34^b^
Compression/coagulation	9 (5.9)	14 (9.2)	NA	
Suture	0	1 (0.7)	NA	
All	9 (5.9)	15 (9.8)	1.72 (0.68-4.63)^c^	.29^d^
Integrity of the staple line	0	0	NA	NA
Incidence of pancreatic injury				
None	149 (98.0)	144 (94.1)	NA	.18^b^
Repair	3 (2.0)	8 (5.2)	NA	
Reresection	0	1 (0.7)	NA	
All	3 (2.0)	9 (5.9)	3.09 (0.75-18.12)^c^	.14^d^
Additional suturing of the stump	2 (1.3)	6 (3.9)	3.05 (0.53-31.38)	.28^d^
Conversion to open surgery	12 (14.1)	12 (13.8)	0.97 (0.37-2.54)	>.99^d^
Drainage duration, median (range), d	6 (1-175)	6 (1-118)	NA	.29^c^
Postoperative hospital stay, median (range), d	16 (4-101)	16 (6-84)	NA	.82^a^
Intra-abdominal hemorrhage				
None	153 (98.7)	152 (98.7)	NA	>.99^b^
Grade A	0	0	NA	
Grade B	0	1 (0.6)	NA	
Grade C	2 (1.3)	1 (0.6)	NA	
All	2 (1.3)	2 (1.3)	1.01 (0.07-14.05)^c^	>.99^d^
Complications	107 (68.6)	101 (65.6)	0.87 (0.53-1.44)	.63^d^
Mortality	0	0	NA	NA
Splenic vein thrombosis				
1 mo After surgery	8 (5.6)	13 (9.2)	1.70 (0.63-4.89)	.27^d^
6 mo After surgery	4 (2.7)	5 (3.6)	1.36 (0.29-7.02)	.74^d^

Abbreviations: NA, not applicable; OR, odds ratio.

^a^ Wilcoxon test.

^b^ Freeman-Halton exact test.

^c^ ORs and 95% CIs were calculated for all events.

^d^ Fisher exact test.

### Additional Analysis

Finally, intraoperative adverse events based on the modified intent-to-treat set are shown in eTable 4 in Supplement 3. The incidence of all adverse events in the combined division group was 3.9% (6 of 154) and that in the separate division group was 2.6% (4 of 155) (odds ratio, 0.65; 95% CI, 0.13-2.82; *P* = .54). No patients experienced grade-4 adverse events.

## Discussion

In this trial, we aimed to establish the safety of combined division compared with separate division of the splenic vein. The proportion of grade B/C PF in the separate division group was 27.1% vs 28.6% in the combined division group, demonstrating noninferiority of the combined division of the splenic vein against separate division. The safety of combined division of the splenic vein in DP was therefore established.

The primary end point result robustly demonstrated noninferiority of the combined division of the splenic vein; furthermore, the intra-abdominal hemorrhage reportedly caused by PF or other reasons in 1% to 8% of patients who undergo DP^2,6,9^ was observed in only 1.3% of the patients in either of the 2 groups in this study. Thus, the results of our analysis of the primary and secondary end points clearly met our hypotheses that dissecting the splenic vein without isolation from the pancreatic parenchyma does not increase the incidence of fatal complications and that the use of mechanical staplers to transect the distal pancreas is feasible. However, there were no differences in operative time or blood loss between the groups, so we could not demonstrate the clinical benefit of the combined division in this regard.

Regarding the secondary end points, the rate of patients requiring hemostasis of the staple line after pancreatic resection was higher in the combined division group vs the separate division group (9.8% vs 5.9%, respectively). The incidence of pancreatic injury also tended to be more frequent in the combined division group vs the separate division group (5.9% vs 2.0%, respectively). Although both results did not reach a statistically significant difference, our results imply that utmost care should be taken and close perioperative observation of the pancreatic stump should be considered mandatory when combined division using mechanical staplers is adopted in DP.

Previous studies reported that pancreatectomy using mechanical staplers often led to postoperative thrombosis in the remnant splenic vein or portal vein after DP.^22,23,24^ In the current trial, the incidence of postoperative splenic vein thrombosis in the combined division group was 9.2%, whereas that in the separate division group was 5.6%. Although the difference was not statistically significant, and the incidence of thrombosis in the current series was not high compared with the incidence in previous reports,^22,23,24^ this result still suggests the possibility that combined division could result in clot formation in the remnant splenic vein. In all cases, careful follow-up after DP is necessary, using postoperative diagnostic imaging. If thrombosis was detected in the splenic or portal veins, anticoagulation therapy should be initiated as soon as possible.

In the current study, because simultaneous division of the pancreatic parenchyma and splenic vein in 1 session was assumed, the distance between the left border of the portal vein and the estimated pancreatic cut end was measured at registration by evaluating preoperative imaging study findings. In fact, combined division close to the confluence of the portal and splenic veins is extremely dangerous because of the risk of damaging the portal vein. In this study, a distance of more than 10 mm was secured so that we completely avoided intraoperative damage to or postoperative stenosis of the portal vein. Sufficient distance must be considered with combined division.

### Limitations

First, it might have been more reasonable to compare the incidence of intra-abdominal hemorrhage as the primary end point. However, we chose to look instead at the incidence of grade B/C PF because hemorrhage was expected to be rare (1%-8%)^2,6,9^ and because our sample size calculation suggested that a study design based on intra-abdominal hemorrhage would require too many patients. However, the main reason for the hemorrhage after DP is thought to be due to the PF. If the rates of PF and subsequent intra-abdominal hemorrhage are comparable between the groups, we supposed that the safety of combined division could be well demonstrated. Hence, this trial was designed to demonstrate the noninferiority of arm B compared with arm A in terms of the incidence of grade B/C PF.

Second, the grade B/C PF incidence rate was originally assumed as 10% for the statistical analyses; however, the actual proportion of PF in the separate division group was 27.1% and that in the combined division group was 28.6%, which were higher proportions than expected. Generally, the PF rate in clinical trials may be worse than the rate in observational studies. Changing the assumption of the grade B/C PF incidence rate from 10% to 27.1% reduces the power in this study from 83.9% to 55.3%. However, the difference of the PF rate was considerably smaller than noninferiority margin 0.09 (modified intent-to-treat set: −0.014; per-protocol set: 0.003). It demonstrated the noninferiority of combined division of the splenic vein compared with separate division of the splenic vein in both the modified intent-to-treat set and the per-protocol set.

## Conclusions

In conclusion, the multicenter COSMOS-DP trial successfully demonstrated the noninferiority of combined division of the splenic vein compared with separate division of the splenic vein in terms of the incidence of grade B/C PF. Thus, the safety of this procedure was established such that it could be recommended with more confidence.

## Notes

Supplement 1. Trial protocol

Supplement 2. eTable 1. Participating Institutes eTable 2. Eligibility Criteria eTable 3. Secondary Endpoint Results for the Per Protocol Set eTable 4. Intraoperative Adverse Events in the Modified Intent-To-Treat (mITT) Set eFigure 1. Intraoperative photograph of the procedure in Arm A (separate division of the splenic vein) eFigure 2. Intraoperative photograph of the procedure in Arm B (combined division of the splenic vein)

Supplement 3. Data sharing statement
