# Supplementary material for: Safety of Combined Division vs Separate Division of the Splenic Vein in Patients Undergoing Distal Pancreatectomy: A Noninferiority Randomized Clinical Trial
Source: JAMA Surg. 2021 Mar 3;156(5):418–28. doi: 10.1001/jamasurg.2021.0108 (PMC7931136; doi:10.1001/jamasurg.2021.0108)
Supplement: Supplement 1. — Trial protocol [file jamasurg-e210108-s001.pdf]

COmbined resection versus **S**eparated resection after **MO**bilization of the **S**plenic vein during **D**istal **P**ancreatectomy; A multicenter, prospective, randomized phase III trial

- COSMOS-DP trial –

Principal Investigator
Hiroki Yamaue
Wakayama Medical University School of Medicine
811-1 Kimiidera, Wakayama 641-8510
TEL: +81-73-441-0612
FAX: +81-73-446-6566
E-mail: yamaue-h@wakayama-med.ac.jp

Ver 0.1 2015/12/30
Ver 0.2 2016/1/7
Ver 0.3 2016/2/29
Ver 0.4 2016/3/17
Ver 0.5 2016/3/29
Ver 1.0 2016/6/13
Ver 2.0 2016/12/5
Ver 2.1 2017/5/22
Ver 2.2 2017/6/5
Ver 2.3 2019/12/3

1. Study purpose

The aim of the COSMOS-DP trial is to establish the non-inferiority of the safety of resecting the splenic vein together with the pancreatic parenchyma compared with that of the conventional technique of isolating the vein from the pancreas before ligation and division during DP using mechanical staplers.

The primary endpoint; the incidence of grade B/C PF
The secondary endpoints;
Outcome measures related to surgery; the operative time, blood loss volume, haemostasis of the staple line, integrity of the staple line, incidence of pancreatic injury, need for additional sutures to securely close the pancreatic stump, drainage duration, postoperative hospital stay duration and incidence of conversion from laparoscopic surgery to open surgery.
Outcome measures related to complications; the incidence of all grades of PF, incidence of grade C PF, incidence of intra-abdominal haemorrhage, incidence of all complications, mortality and the incidence of splenic vein thrombosis (1 and 6 months after surgery)

2. Backgrounds

In general, in distal pancreatectomy (DP) involves not only mandatory dissection of the pancreas but also dissection of the splenic artery and vein. During this surgical procedure, the splenic vein is isolated from the pancreatic parenchyma prior to being ligated and divided. The reason for this is to prevent intra-abdominal haemorrhage from the stump of the splenic vein following the occurrence of pancreatic fistula (PF), which is commonly observed after DP (8.6-42.3%). Although it is relatively easy to isolate the splenic vein at the confluence of the portal and splenic veins, it is often firmly embedded in the pancreatic parenchyma at more distal regions of the pancreas. Furthermore, the splenic vein can be difficult and time-consuming to isolate due to the necessity of carefully handling all small branches that flow from the parenchyma to the splenic vein. Thus, at times, this procedure can lead to hardships, as well as an unexpectedly large volume of blood loss.

More recently, mechanical staplers have been increasingly used to dissect the pancreas, particularly when DP is performed using a laparoscopic approach. Under such circumstances, the splenic vein is often dissected together with the pancreatic parenchyma, with no attempt of isolation. This method of pancreatic dissection has become the standard at some institutions and has been reputed for its apparent technical simplicity[1-3]. However, PF occurring after this type of resection is of deep concern to surgeons due to the risk of intra-abdominal bleeding from the stump of the splenic vein, which could then be immersed in effusion rich in pancreatic juice.

To date, no scientific investigation of the safety of this useful but potentially hazardous surgical procedure has been performed. Therefore, we plan to conduct a prospective

randomized study to establish the safety of this procedure so that it can be recommended with more confidence. The use of this procedure will likely result in significant reductions in operative time and blood loss during DP.

3. Study design

1) Objective

Patients undergoing open or laparoscopic DP for pancreatic body and tail cancer, intra-ductal papillary mucinous neoplasm, neuroendocrine tumours, mucinous cystic neoplasm, or metastatic pancreatic tumours or similar are eligible for inclusion in this study. In addition, simultaneous resection of the pancreatic parenchyma and splenic vein in one session will be rendered possible through the evaluation of preoperative imaging study findings.

2) Study design

This study is designed as a multicentre (45 institutes) prospective randomized phase III trial.

3) Arms

Arm A (separate resection of the splenic vein)
Arm B (combined resection of the splenic vein)

4) Randomization

Central randomization and registration system will be applied (1:1). Upon randomization, the patients will be stratified by the surgical approach used (open or laparoscopic), institution and thickness of the pancreatic parenchyma (<15 mm or ≥15 mm)

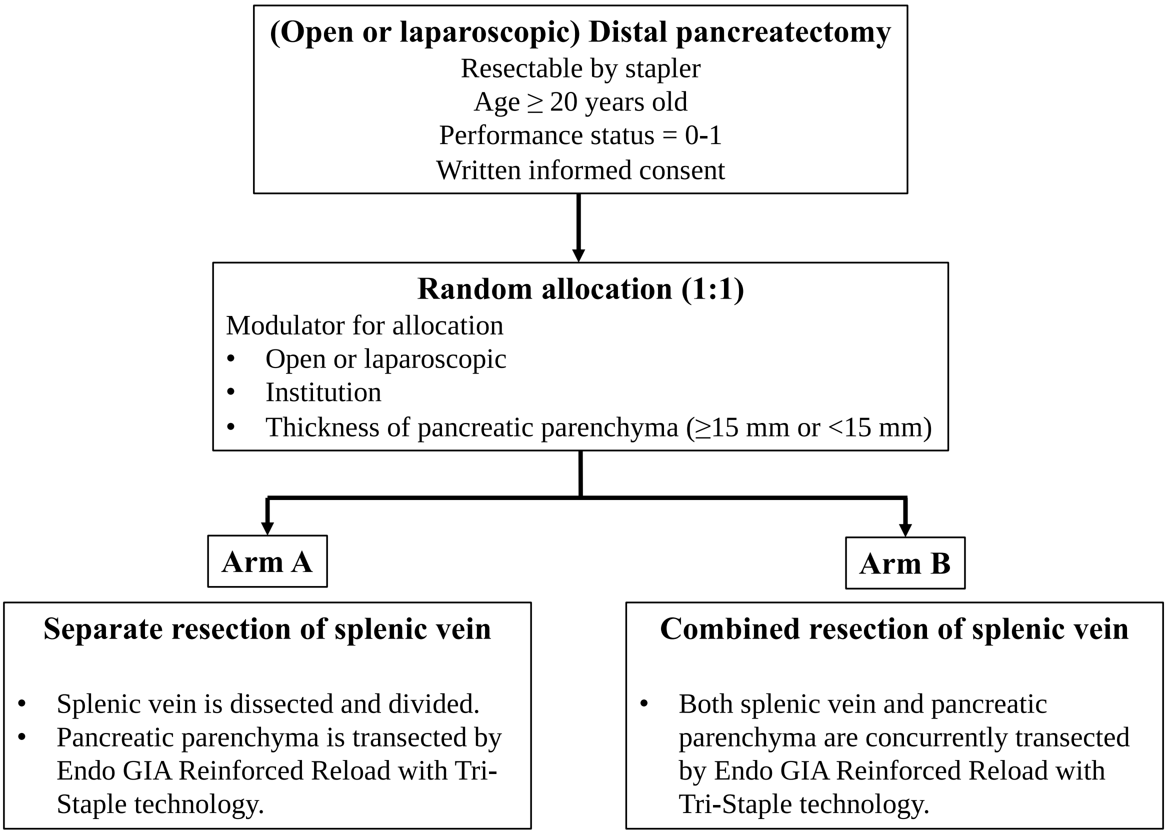

5) Sample size

304 patients（Arm A：152 patients，Arm B：152 patients）

6) Statistical analysis

This trial was designed to evaluate the non-inferiority of group B compared with group A in terms of the incidence of PF grade B/C. The incidence of clinically relevant PF (grade B/C) after DP using a stapler has been reported to be 1.9-20.3% in recent clinical trials[4-6]. Therefore, for an assumed PF incidence rate of 10% with a non-inferiority margin of 9%, the difference of allowable PF incidence rate between groups A and B is 0.09, and the allowable odds ratio is 2.11. When the statistical analysis is performed for a significance level of α = 0.05 (one side) in a non-inferiority design, 138 patients are calculated to be required per arm, with a power 100 (1-β) of more than 80%, under the assumption that a small number of patients may be deemed ineligible and may thus be excluded from the analysis. Furthermore, as approximately 5% of the patients are expected to be ineligible for surgery as indicated by the laparotomy or laparoscopic findings, the sample size was eventually increased to 304 patients (152 patients per arm).

7) Enrollment and follow-up period

- Enrollment period：IRB approval-2019/7/31（3 years）
- Follow-up period：6 months after final enrollment
- Total study period：IRB approval-2020/7/31

4．Study population

1) Objective

Patients undergoing open or laparoscopic DP for pancreatic body and tail cancer, intra-ductal papillary mucinous neoplasm, neuroendocrine tumours, mucinous cystic neoplasm, or metastatic pancreatic tumours or similar are eligible for inclusion in this study.

2) Operative procedure

- Open or laparoscopic DP
- Simultaneous resection of the pancreatic parenchyma and splenic vein in one session will be rendered possible through the evaluation of preoperative imaging study findings.
- Patients indicated for spleen-preserving distal pancreatectomy (SPDP) will be excluded.
- The Warshaw operation (spleen-preserving and splenic artery/vein resection) will be included.
- The extent of lymph node dissection and whether the celiac axis is dissected will be left to the discretion of the surgeons.

3) Inclusion and exclusion criteria

**Inclusion criteria**	**Exclusion criteria**
(i) Elective open or laparoscopic distal pancreatectomy for diseases of the pancreatic body and tail	(i) Splenic vein-preserving distal pancreatectomy<br>(ii) Superior mesenteric vein or portal vein invasion
(ii) ECOG Performance Status (PS) = 0-1	(iii) Pancreatic trauma
(iii) Age ≥ 20 years old	(iv) Preoperative inflammatory pancreatic disease (pancreatitis)
(iv) Maintenance of function of the major organs (bone marrow, liver, kidney, lung, etc.)	(v) Requirement of anti-coagulant treatment during or after surgery*
(a) White blood cells ≥ 2,500/mm^3	(vi) Severe ischemic cardiovascular disease
(b) Haemoglobin ≥ 9.0 g/dL	(vii) Liver cirrhosis or active hepatitis
(c) Platelets ≥ 100,000/mm^3<br>(d) Total bilirubin ≤ 2.0 mg/dL	(viii) Need for oxygen due to interstitial pneumonia or lung fibrosis
(e) Creatinine ≤ 2.0 mg/dL	(ix) Dialysis due to chronic renal failure
(v) Sufficient understanding of the study to	(x) Need for surrounding organ resection

provide written informed consent	(stomach, colon, etc.), excluding the left adrenal gland and gall bladder<br>(xi) Active multiple cancer that is thought to influence the occurrence of adverse events<br>(xii) Difficulty with study participation due to psychotic disease or symptoms<br>(xiii) When a surgeon considers the use of stapler as inappropriate<br>(xiv) Inappropriate for the study objectives

* Anti-coagulant treatment at 24 hrs after surgery is allowed.

5. Protocol treatment

1) Surgical resection

Before pancreatic transection, concurrent division of the splenic artery using a mechanical stapler will not be permitted. A linear stapler, Endo GIA Reinforced Reload with Tri-Staple Technology (Black Cartridge, Covidien®), will be used in all patients. The pancreatic parenchyma will be compressed with the stapler at the planned line of resection for over 5 minutes before transection is performed[7,8]. For the patients in Arm A, the splenic vein will be isolated from the pancreatic parenchyma and dissected after ligation. For those in Arm B, the splenic vein will be transected concurrently with the pancreatic parenchyma using the aforementioned stapler. Anti-coagulant treatment, such as low-molecular-weight heparin or fondaparinux, will be permitted at 48 hrs after surgery.

Antibiotics, plasma expanders, blood products, analgesic drugs, H2 blockers, and proton pump inhibitors will be used for intra- and postoperative management at the discretion of the surgeons. In addition, there are no regulations on the drugs used to control complications and adverse events. Prophylactic administration of octreotide will not be permitted.

2) Intraoperative photography

To confirm that the surgical procedures are conducted as allocated at the time of central judgement, two photographs (before and after pancreatic transection) will be taken for all patients. Central judgement will be conducted biannually for all of the registered patients. At that time, the photographs will be reviewed by more than two members of the committee.

3) Chemotherapy and radiotherapy

There are no regulations regarding the use of preoperative, intraoperative or postoperative chemotherapy or radiotherapy as treatments in this study. However, details including the treatment regimen and cycles given of the preoperative treatment are to be

reported in the case report form.

4) Follow-up after surgery

The presence of thrombus in the splenic vein will be evaluated at 1 and 6 months after surgery by enhanced computed tomography or magnetic resonance imaging.

6. Study management

1) Monitoring

Central monitoring will be performed each year by the data centre to evaluate the study progress and ensure for study quality. The following aspects will be monitored: i) data accumulation; ii) patient eligibility; iii) severe adverse events; iv) protocol deviations; v) reasons for cessation or expiration of the protocol; vi) background factors of the patients; and vii) other problems concerning study progress and safety.

2) Audit

The person appointed by the audit office visits the participating institution and confirms the study implementation system, and whether the study is conducted in a ccordance with the study protocol and the ethical guidelines.

7. Ethical considerations

All investigators will conduct this study according to the Helsinki Declaration.

1) Informed Consent

All subjects for this study will be provided a consent form describing this study and providing sufficient information for subjects to make an informed decision about their participation in this study. The consent form will be submitted with the protocol for review and approval by the Institutional Review Board (IRB) for the study. The formal consent of a subject, using the IRB-approved consent form, must be obtained before that subject is submitted to any study procedure. This consent form must be signed by the subject, and the investigator-designated research professional obtaining the consent. A copy of the IRB-approved form must be kept on-site and by administrative office and data center.

2) IRB review

This protocol and any amendments will be submitted to a properly constituted independent IRB, in agreement with local legal prescriptions, for formal approval of the study conduct. The decision of the IRB concerning the conduct of the study will be made in writing to the investigator and a copy of this decision will be provided to administrative office and data center before commencement of this study.

8. Registration and Allocation

1） Facility registration

Approval of this protocol by the IRB of each institution is required. The research director of each institution send a copy of the approval letter of the IRB and a facility registration form by FAX to the administrative office (Wakayama Medical University).

2） Informed consent

The formal consent of a subject, using the IRB-approved consent form, must be obtained before that subject is submitted to any study procedure. This consent form must be signed by the subject.

3） Case registration

The investigator at each institution confirms the inclusion and exclusion criteria before surgery, and then registers the subject of the data center.

4） Registration number

After confirming that the conditions are satisfied, the registration number is issued and "registration" is completed at this point. If the input data is inadequate or does not meet the eligibility criteria, the registration number will not be issued and it will not be "registered".

5） Allocation

When the case registration is made, the assigned group (A group or B group) and "registration notice form" showing the registration number are displayed. The investigator shall start treatment after completion of registration.

6） Start of the protocol treatment

The research director or the investigator will start the protocol treatment within 28 days after registration after confirming the completion of the case registration with "registration notice form". If it is ineligible, the investigator explains to the subject that registration to this study is not possible.

Registration after the start of protocol treatment is not allowed without exception

9. Examination and observation parameters

1） Preoperative parameters

A) Hight/Body weight/BMI
B) DM/Steroid use/Anticoaglant/Cardiac disorder
C) Preoperative treatment（chemotherapy・chemoradiotherapy）
D) Blood examination（WBC/Hb/PLT/T-Bil/Cre/Alb/Amy/HbA1c（NGSP））

E) Pancreatic thickness（Preoperative CT or MRI imaging）

F) MPD diameter

G) Original disease

2）Operative parameters

A) Operative procedure（Open or laparoscopic/DP-CAR/SPDP）

B) Conversion to open surgery from laparoscopic surgery

C) Lymph node dissection（D0-D3）

D) Concurrent arterial resection（celiac artery）

E) Operative time（min）

F) Blood loss（ml）

G) Blood transfusion（RBC/FFP）

H) Pancreatic texture（soft/hard）

I) Pancreatic cut line（Preoperative CT or MRI）

J) Cartridge number

K) Reinforcement sheet (coated/non-coated)

L) Drainage number and site

M) Intraoperative parameters of stapler
haemostasis of the staple line, integrity of the staple line, incidence of pancreatic injury, need for additional sutures to securely close the pancreatic stump

N) Other staple-related adverse events

Score	Integrity of the staple line	Haemostasis of the staple line	Pancreatic injury
0	No	No	No
1	Yes	Compression or coagulation	Injury for repair
2		Suture	Need for re-resection

3）Postoperative parameters

A) POD1/3/7 Blood examination（WBC/Hb/Alb/Amy/CRP）

B) POD1/3 Drain amylase

C) Postoperative complications

D) POPF grade/POH/Mortality/Thrombosis or Obstruction of splenic vein（1 month/6 months）

E) Postoperative course

- Drainage period
- Postoperative hospital stay

4) Discontinuation parameters

- Reason

Study calendar

	Pre-		Intra-	Post-		
	Pre-registration	Post-registration		Until discharge	1 month after surgery	6 months after surgery
Informed consent	●					
Registration	●					
Patient background	●	●				
Blood examination	●	●		●		
Operation			●			
Drainage amylase level				●		
Drainage removal				●		
Adverse events			←	—	—	→
CT/MRI	●				●	●

10. Definition of complications

Defined as an event that occurred within 6 months after surgery

1） Postoperative pancreatic fistula (POPF)
International Study Group of Postoperative Pancreatic Fistula（ISGPF）
Bassi C, Dervenis C, Butturini G, et al. Postoperative pancreatic fistula: An international study group (ISGPF) definition. Surgery 2005; 138:8-13.

2） Delayed gastric emptying (DGE)
International Study Group of Pancreatic Surgeons (ISGPS)
Wente MN, Bassi C, Dervenis C, et al. Delayed gastric emptying (DGE) after pancreatic surgery: a suggested definition by the International Study Group of Pancreatic Surgery (ISGPS). Surgery 2007;142:761-768.

3） Postpancreatecomy hemorrhage (PPH)

International Study Group of Pancreatic Surgeons (ISGPS)
Wente MN, Veit JA, Bassi C, et al. Postpancreatectomy hemorrhage (PPH)-An International Study Group of Pancreatic Surgery (ISGPS) definition. Surgery 2007:142:0-25.

4） Other complications
Clavien classification
Grade I: any deviation from the normal postoperative course but without any need for pharmacologic treatment or surgical, endoscopic, or radiologic intervention
Grade II: Requiring pharmacological treatment with drugs other than such allowed for grade I complications. Blood transfusions and total parenteral nutrition are also included.
Grade III: complications requiring surgical, endoscopic, or radiologic intervention
IIIa: intervention not under general anesthesia
IIIb: intervention under general anesthesia
Grade IV: life-threatening complications requiring intermediate or intensive care unit (ICU) management
IVa: single organ dysfunction (including dialysis)
IVb: multi organ dysfunction
Grade V: death
Suffix 'd': If the patients suffers from a complication at the time of discharge, the suffix “d” (for ‘disability’) is added to the respective grade of complication. This label indicates the need for a follow-up to fully evaluate the complication.
Dindo D, Demartines N, Clavien PA. Classification of surgical complications: a new proposal with evaluation in a cohort of 6336 patients and results of a survey. Ann Surg. 2004;240:205-13.

11. Report of adverse events

1. Report of adverse events
If "serious adverse event" or "unexpected adverse event" occurs, the research director of the institution reports to the administrative office (Wakayama Medical University).
1) Leading to death
2) Life threatening
3) Requiring hospitalization or prolongation of hospital stay
4) Leading to everlasting or serious malfunction
5) Leading to congenital malformation or deficiency
2. Expedited report of adverse events
1) All deaths "during protocol treatment" or "within 30 days after protocol treatment"
2) Unexpected Grade IV adverse events
An adverse event that is judged to be causally related to protocol treatment shall be subject to expedited report.

If an adverse event to be subject to expedited report occurs, the investigator shall report to Wakayama Medical University by e-mail within 48 hours. Wakayama Medical University distributes emails about this adverse events to the research director of each institution to share information. The principle investigator will report to the Efficacy and Safety Evaluation Committee as soon as possible, and ask the judge about the adequacy of response to the adverse event.

3. Normal report of adverse events

1) Deaths after 31 days from the end of protocol treatment

Deaths suspected of treatment related death are applicable. Obvious original disease death does not apply.

2) Expected Grade IV adverse events

3) Unexpected Grade III adverse events

4) Other serious medical events

12. Definition of endpoints

Primary endpoint

1） Incidence of a clinically relevant POPF (ISGPF grade B/C)

Incidence of a clinically relevant POPF (grade B/C), according to the ISGPF criteria which is the evaluation criteria for POPF

Secondary endpoints

1） Operative time

Time from start of surgery to end of surgery

2） Blood loss

Total blood loss during surgery

3） Preoperative pancreatic thickness

Pancreatic thickness of estimated pancreatic cut end measured by preoperative CT or MRI

4） Incidence of bleeding from staple line

Incidence to need additional hemostasis (including coagulation or suture) for bleeding of staple line after pancreatectomy of mechanical stapler

5） Grade of bleeding from staple line

Grade to need additional hemostasis (including coagulation or suture) for bleeding of staple line after pancreatectomy of mechanical stapler

6） Incidence of integrity of the staple line

Incidence of insufficient suture on staple line line after pancreatectomy of mechanical stapler

7） Incidence of pancreatic injury

Incidence of pancreatic injury on staple line during or after pancreatectomy of mechanical stapler

8） Grade of pancreatic injury

Grade of pancreatic injury on staple line during or after pancreatectomy of mechanical stapler

9） Incidence of additional suture for pancreatic stump

Incidence to need additional suture of staple line for hemostasis or repair after pancreatectomy of mechanical stapler

10）Length of drain placement

Number of days from operation date to drain removal date (the peripancreatic drain to be removed last)

11）Length of the hospital stay

Number of days from operation date to discharge date

12）Incidence of conversion from laparoscopic surgery to open surgery

Incidence of conversion to open surgery during laparoscopic surgery

13）Incidence of overall POPF (Grade A, grade B, and C)

Incidence of overall POPF of grade A, grade B, or grade C, according to the ISGPF criteria

14）Incidence of POPF grade C

Incidence of POPF grade C, according to the ISGPF criteria which is the evaluation criteria for POPF

15）Incidence of postpancreatectomy hemorrhage (PPH)

Incidence of overall PPH, according to the ISGPS criteria

16）Incidence of overall postoperative complications

Incidence of overall postoperative complications, according to Clavien-Dindo classification

17）Incidence of 90-day mortality

Incidence of surgery-related deaths from operation date to postoperative 3 months

18）Incidence of thrombosis of splenic vein

Incidence of thrombosis of splenic vein by follow-up CT or MRI after 1month or 6 months after surgery

13. Modification, discontinuation, and completion of the study

1) Modification of the protocol

When contents of the protocol are modified, the principal investigator submits contents of all modifications to IRB of Wakayama Medical University, and reports them to research director of each institution immediately after approval. The research director of each institution obtains approval from IRB of each institution.

2) Discontinuation of the study

If the study is discontinued or suspended, the principal investigator must notify research director of each institution, investigators, data center, Efficacy and Safety Assessment Committee, and the persons concerned immediately. Investigators must notify subjects immediately.

3) Completion of the study

If the study is completed, the investigator will notify research director of each institution, investigators, data center, Efficacy and Safety Assessment Committee, and the persons concerned immediately. The research director of each institution will immediately report its completion to relevant departments of the institution.

14. Financing

This study is funded by Wakayama Medical University. The status of conflicts of interest of the principle investigator is examined by the Conflicts of Interest Management Committee of Wakayama Medical University, prior to the ethical review by the IRB.

15. Organization

1) Principal Investigator

Hiroki Yamaue
Wakayama Medical University School of Medicine
811-1 Kimiidera, Wakayama 641-8510
TEL: +81-73-441-0612
FAX: +81-73-446-6566
E-mail: yamaue-h@wakayama-med.ac.jp

2) Study office

Tsutomu Fujii
Department of Surgery and Science, Faculty of Medicine, Academic Assembly, University of Toyama
2630, Sugitani, Toyama 930-0194 Japan

TEL: +81-76-434-7331
FAX: +81-76-434-5043
E-mail: fjt@med.u-toyama.ac.jp

Suguru Yamada
Nagoya University Graduate School of Medicine, Gastroenterological Surgery (Surgery II)
65 Tsurumai-cho, Showa-ku, Nagoya, Aichi 466-8550, Japan
TEL: +81-52-744-2245
FAX: +81-52-744-2255
E-mail: suguru@med.nagoya-u.ac.jp

3) Registration/Data center/Monitoring office
Megumi Kitayama
Wakayama Medical University School of Medicine
811-1 Kimiidera, Wakayama 641-8510
TEL: +81-73-441-0878
FAX: +81-73-441-0868
E-mail: dc_csc@wakayama-med.ac.jp

4) Protocol committee
（Chairman）Wakayama Medical University Hiroki Yamaue
Osaka University Hidetoshi Eguchi
Kansai Medical University Sohei Satoi
Kyushu University Masafumi Nakamura
Tokyo Medical University Yuichi Nagakawa
University of Toyama Tsutomu Fujii
Hiroshima University Yoshiaki Murakami
Wakayama Medical University Toshio Shimokawa
Wakayama Medical University Manabu Kawai

5) Data management/Registration/Monitoring
Wakayama Medical University Megumi Kitayama

6) Randomization
Wakayama Medical University Toshio Shimokawa

7) Statistical analysis
Wakayama Medical University Toshio Shimokawa

8) Efficacy and safety assessment committee

Akita University Yuzo Yamamoto
Ehime University Yasutsugu Takada
Shimane University Yoshitsugu Tajima

9) Operative procedure assessment committee
Kagawa University Yasuyuki Suzuki
Shiga Medical University Shinji Tani

10) Audit
CLINICAL STUDY SUPPORT
TEL: +81-52-211-2011
FAX: +81-52-211-2012

16. References

1. Uyama I, Sugioka A, Sakurai Y, Komori Y, Hanai T, Matsui H, et al. Hand-assisted laparoscopic function- preserving and radical gastrectomies for advanced-stage proximal gastric cancer. J Am Coll Surg. 2004;199(3):508-15.
2. Shimizu S, Tanaka M, Konomi H, Mizumoto K, Yamaguchi K. Laparoscopic pancreatic surgery: current indications and surgical results. Surg Endosc. 2004;18(3):402-6.
3. Misawa T, Shiba H, Usuba T, Nojiri T, Uwagawa T, Ishida Y, et al. Safe and quick distal pancreatectomy using a staggered six-row stapler. Am J Surg. 2008;195(1):115-8.
4. Olah A, Issekutz A, Belagyi T, Hajdu N, Romics L, Jr. Randomized clinical trial of techniques for closure of the pancreatic remnant following distal pancreatectomy. Br J Surg. 2009;96(6):602-7.
5. Diener MK, Seiler CM, Rossion I, Kleeff J, Glanemann M, Butturini G, et al. Efficacy of stapler versus hand-sewn closure after distal pancreatectomy (DISPACT): a randomised, controlled multicentre trial. Lancet. 2011;377(9776):1514-22.
6. Carter TI, Fong ZV, Hyslop T, Lavu H, Tan WP, Hardacre J, et al. A dual-institution randomized controlled trial of remnant closure after distal pancreatectomy: does the addition of a falciform patch and fibrin glue improve outcomes? J Gastrointest Surg. 2013;17(1):102-9.
7. Nakamura M, Ueda J, Kohno H, Aly MY, Takahata S, Shimizu S, et al. Prolonged peri-firing compression with a linear stapler prevents pancreatic fistula in laparoscopic distal pancreatectomy. Surg Endosc. 2011;25(3):867-71.
8. Hamilton NA, Porembka MR, Johnston FM, Gao F, Strasberg SM, Linehan DC, et al. Mesh reinforcement of pancreatic transection decreases incidence of pancreatic occlusion failure for left pancreatectomy: a single-blinded, randomized controlled trial. Ann Surg. 2012;255(6):1037-42.
