# Supplementary material for: Safety of Combined Division vs Separate Division of the Splenic Vein in Patients Undergoing Distal Pancreatectomy: A Noninferiority Randomized Clinical Trial
Source: JAMA Surg. 2021 Mar 3;156(5):418–28. doi: 10.1001/jamasurg.2021.0108 (PMC7931136; doi:10.1001/jamasurg.2021.0108)
Supplement: Supplement 2. — eTable 1. Participating Institutes eTable 2. Eligibility Criteria eTable 3. Secondary Endpoint Results for the Per Protocol Set eTable 4. Intraoperative Adverse Events in the Modified Intent-To-Treat (mITT) Set eFigure 1. Intraoperative photograph of the procedure in Arm A (separate division of the splenic vein) eFigure 2. Intraoperative photograph of the procedure in Arm B (combined division of the splenic vein) [file jamasurg-e210108-s002.pdf]

Supplemental Online Content

Yamada S, Fujii T, Sonohara F, et al. Safety of combined division vs separate division of the splenic vein in patients undergoing distal pancreatectomy: a noninferiority randomized clinical trial. *JAMA Surg*. Published online March 3, 2021.
doi:10.1001/jamasurg.2021.0108

eTable 1. Participating Institutes
eTable 2. Eligibility Criteria
eTable 3. Secondary Endpoint Results for the Per Protocol Set
eTable 4. Intraoperative Adverse Events in the Modified Intent-To-Treat (mITT) Set
eFigure 1. Intraoperative photograph of the procedure in Arm A (separate division of the splenic vein)
eFigure 2. Intraoperative photograph of the procedure in Arm B (combined division of the splenic vein)

This supplemental material has been provided by the authors to give readers additional information about their work.

© 2021 Yamada et al. *JAMA Surgery*.

eTable 1. Participating Institutes

1. Nagoya University Hospital
2. Wakayama Medical University Hospital
3. Kumamoto University Hospital
4. Kinki University Hospital
5. Tohoku University Hospital
6. National Defence Medical College Hospital
7. Showa University Hospital
8. Bantane Hotokukai Hospital
9. Nara Medical University Hospital
10. Keio University Hospital
11. Toho University, Omori Hospital
12. Kansai Medical University Hospital
13. The Hospital of Hyogo College of Medicine
14. Oita Red Cross Hospital
15. Toyama Prefectural Central Hospital
16. Fukuyama Medical Center
17. Sendai Kousei Hospital
18. Kyushu University Hospital
19. Kyoto Katsura Hospital
20. St. Marianna University School of Medicine Hospital
21. Kansai Rosai Hospital
22. Tokyo Medical And Dental University Hospital Faculty of Medicine
23. University Hospital Kyoto Prefectural University of Medicine
24. Osaka University Hospital
25. Toyama University Hospital
26. Hiroshima University Hospital
27. Oita University Hospital
28. Sapporo Medical University Hospital
29. University of Miyazaki Hospital
30. Gifu University Hospital
31. Kyoto University Hospital
32. Japanese Red Cross Otsu Hospital
33. Fukushima Medical University Hospital
34. Saiseikai Yokohamashi Tobu Hospital
35. Osaka City University Hospital
36. Hiroshima City Hiroshima Citizens Hospital
37. Japanese Red Cross Musashino Hospital
38. University of Yamanashi Hospital
39. Hokkaido University Hospital
40. Tokyo Medical University Hospital
41. Teikyo University Medical Center
42. Aichi Cancer Center
43. Kurume University Hospital
44. Yamaguchi University Hospital
45. Osaka City General Hospital

© 2021 Yamada et al. *JAMA Surgery*.

eTable 2. Eligibility Criteria

Inclusion Criteria	Exclusion Criteria
(i) Elective open or laparoscopic distal pancreatectomy for diseases of the pancreatic body and tail<br>(ii) ECOG Performance Status (PS) = 0-1<br>(iii) Age ≥20 years<br>(iv) Maintained function of the major organs (bone marrow, liver, kidney, lung.)<br>(a) White blood cells ≥2500 cells/mm^3<br>(b) Hemoglobin ≥9·0 g/dL<br>(c) Platelets ≥100,000/mm^3<br>(d) Total bilirubin ≤2·0 mg/dL<br>(e) Creatinine ≤2·0 mg/dL<br>(v) Sufficient understanding of the study to provide written informed consent	(i) Splenic vein-preserving distal pancreatectomy<br>(ii) Superior mesenteric vein or portal vein invasion<br>(iii) Pancreatic trauma<br>(iv) Preoperative inflammatory pancreatic disease (pancreatitis)<br>(v) Requirement for anti-coagulant treatment during or after surgery*<br>(vi) Severe ischemic cardiovascular disease<br>(vii) Liver cirrhosis or active hepatitis<br>(viii) Need for oxygen due to interstitial pneumonia or lung fibrosis<br>(ix) Dialysis due to chronic renal failure<br>(x) Need for surrounding organ resection (stomach, colon.), excluding the left adrenal gland and gall bladder<br>(xi) Active multiple cancers considered to influence the occurrence of adverse events<br>(xii) Difficulty with study participation due to psychiatric disease or symptoms<br>(xiii) When a surgeon considered the use of a stapler as inappropriate<br>(xiv) Inappropriate for the study objectives

ECOG=Eastern Cooperative Oncology Group
*Anti-coagulant treatment 24 h after surgery was allowed.

© 2021 Yamada et al. *JAMA Surgery*.

eTable 3. Secondary Endpoint Results for the Per Protocol Set

	Separate Division n=146	Combined Division n=133	OR (95% CI)	p Value
**Secondary Endpoints**				
Operative time (min)	246 (106–533)	247 (80–612)	··	0.868[b]
Blood loss (ml)	100 (0–1612)	95 (0–3170)	··	0.651[b]
Hemostasis of the staple line				
None	137 (93.8%)	119 (89.5%)	··	0.226[c]
Compression/coagulation	9 (6.2%)	13 (9.8%)	··	
Suture	0 (0.0%)	1 (0.8%)	··	
All	9 (6.2%)	14 (10.5%)	1.79 (0.69–4.86)[d]	0.199[a]
Integrity of the staple line	0 (0.0%)	0 (0.0%)	··	··
Incidence of pancreatic injury				
None	143 (97.9%)	125 (94.0%)	··	0.124[c]
Repair	3 (2.1%)	8 (6.0%)	··	
Re-resection	0 (0.0%)	0 (0.0%)	··	
All	3 (2.1%)	8 (6.0%)	3.04 (0.71–18.17)[d]	0.124[a]
Additional suturing of the stump	2 (1.4%)	5 (3.8%)	2.80 (0.45–29.91)	0.264[a]
Conversion to open surgery	10 (12.3%)	9 (11.7%)	0.94 (0.32–2.75)	1.000[a]
Drainage duration (days)	6 (1–175)	6 (1–75)	··	0.483[b]
Postoperative hospital stay (days)	16 (4–101)	16 (6–77)	··	0.834[b]
Intra-abdominal hemorrhage				
None	144 (98.6%)	129 (99.2%)	··	0.353[c]
Grade A	0 (0.0%)	0 (0.0%)	··	
Grade B	0 (0.0%)	1 (0.8%)	··	
Grade C	2 (1.4%)	0 (0.0%)	··	
All	2 (1.4%)	1 (0.8%)	0.56 (0.01–10.86)[d]	1.000[a]
Complications	99 (67.8%)	88 (67.7%)	0.99 (0.58–1.70)	1.000[a]
Mortality	0 (0.0%)	0 (0.0%)	··	··
Splenic vein thrombosis				
1 month after surgery	8 (5.9%)	13 (10.4%)	1.85 (0.68–5.36)	0.254[a]
6 months after surgery	4 (2.9%)	4 (3.3%)	1.17 (0.21–6.44)	1.000[a]

Data are expressed as median (range) or n (%). OR=odds ratio. CI=confidence interval.
[a]Fisher's exact test. [b]Wilcoxon test. [c] Freeman-Halton's exact test. [d] ORs and 95%CIs were calculated for all events.

© 2021 Yamada et al. *JAMA Surgery*.

eTable 4. Intraoperative Adverse Events in the Modified Intent-To-Treat (mITT) Set

	Separate Division n=155	Combined Division n=154	OR (95% CI)	p Value
**Intraoperative Adverse Events**				
All	4 (2.6%)	6 (3.9%)	0.65 (0.13–2.82)	0.541[a]
≥G3	4 (2.6%)	3 (1.9%)	1.33 (0.22–9.25)	1.000[a]
G4	0 (0.0%)	0 (0.0%)	··	··

Data are expressed as n (%). OR=odds ratio. CI=confidence interval. G=grade
[a]Fisher's exact test.

© 2021 Yamada et al. *JAMA Surgery*.

eFigure 1.
Arm A (Separate division of the splenic vein)

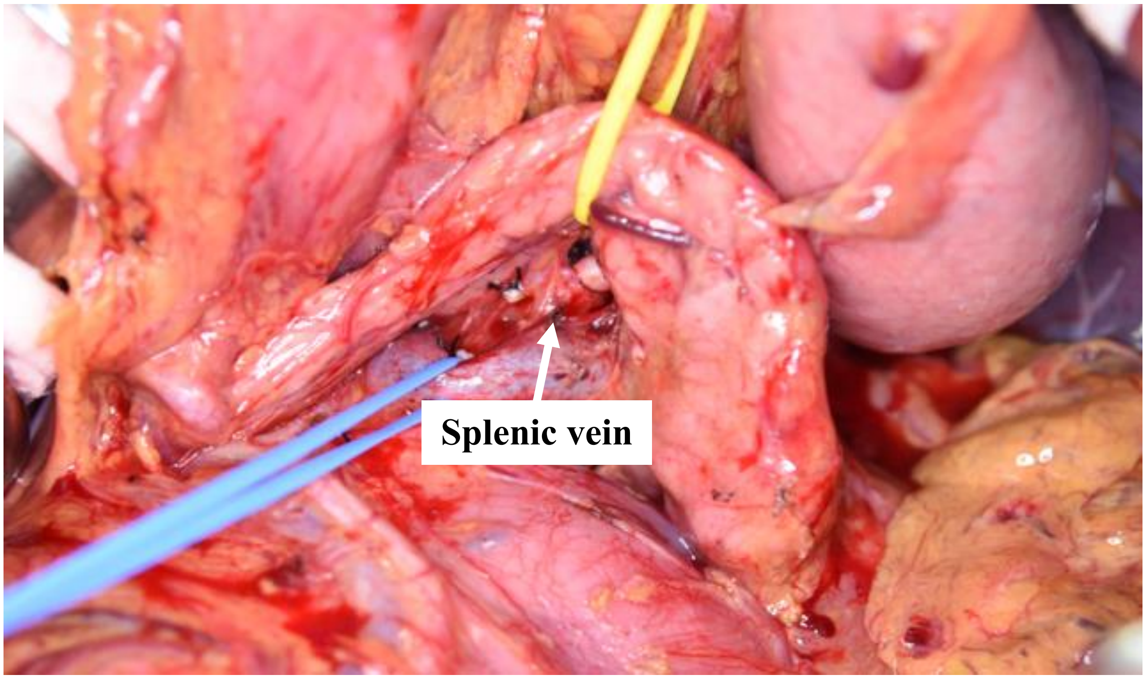

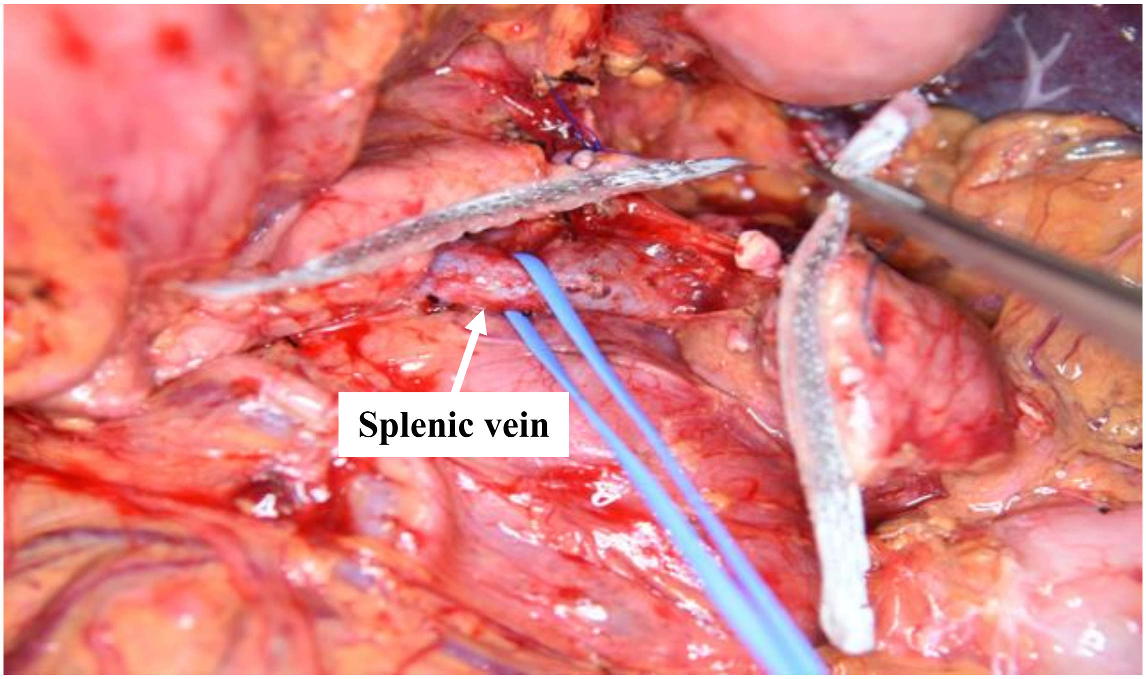

© 2021 Yamada et al. *JAMA Surgery*.

eFigure 2.
Arm B (Combined division of the splenic vein)

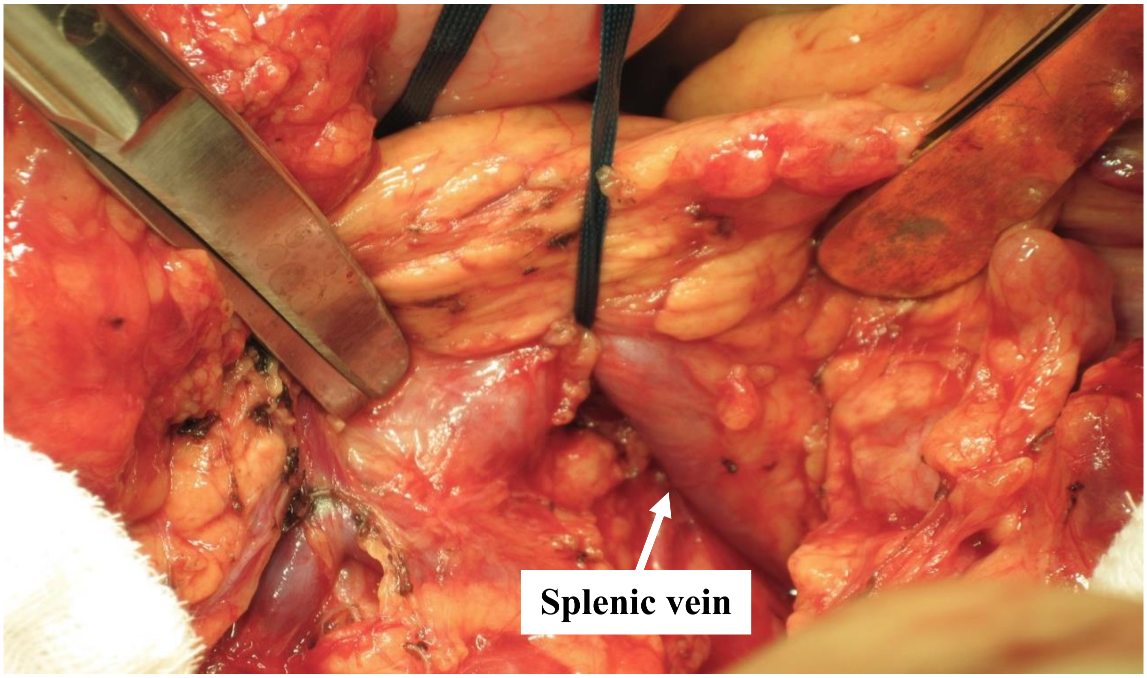

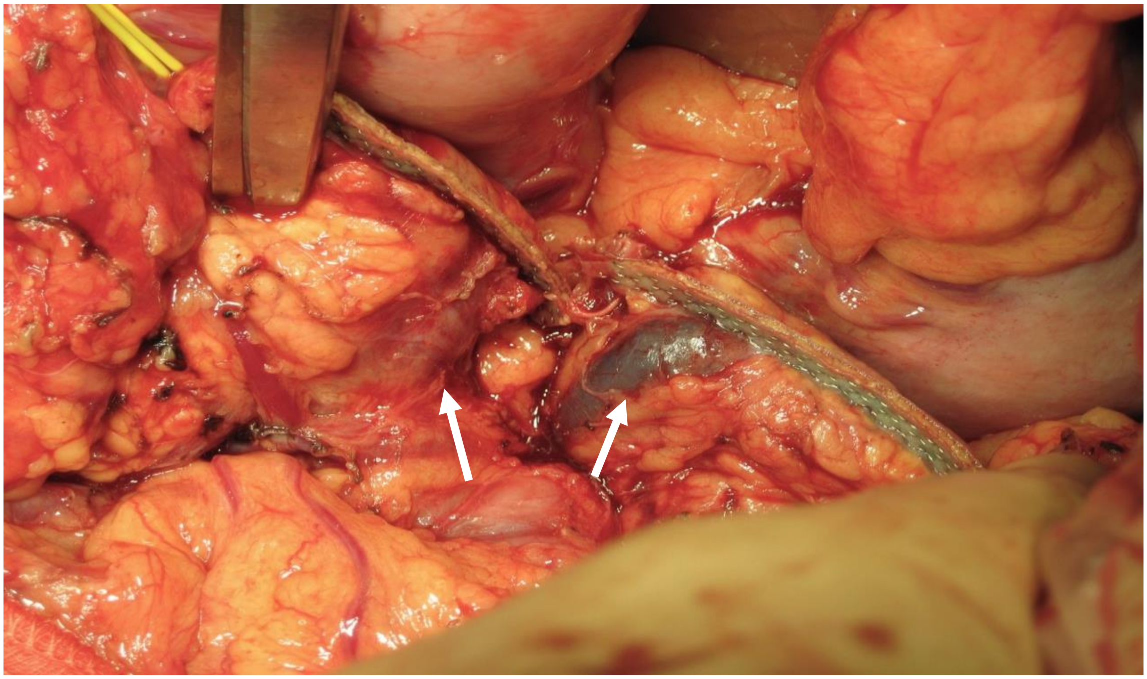

© 2021 Yamada et al. *JAMA Surgery*.
