# Supplementary material for: Safety of Combined Division vs Separate Division of the Splenic Vein in Patients Undergoing Distal Pancreatectomy: A Noninferiority Randomized Clinical Trial
Source: JAMA Surg. 2021 Mar 3;156(5):418–28. doi: 10.1001/jamasurg.2021.0108 (PMC7931136; doi:10.1001/jamasurg.2021.0108)
Supplement: Supplement 3. — Data sharing statement [file jamasurg-e210108-s003.pdf]

Data Sharing Statement

Yamada. Safety of Combined Division vs Separate Division of the Splenic Vein in Patients Undergoing Distal Pancreatectomy. *JAMA Surg*. Published March 03, 2021. doi:10.1001/jamasurg.2021.0108

Data
Data available: Yes
Data types: Deidentified participant data
How to access data: All requests should be submitted to the corresponding author for consideration. (yamaue-h@wakayama-med.ac.jp)
When available: With publication

Supporting Documents
Document types: None

Additional Information
Who can access the data: Researchers whose proposed use of the data has been approved
Types of analyses: For a specified purpose, meta-analysis
Mechanisms of data availability: After approval of a proposal
